# HOSPICE UTILIZATION AND PLACE OF DEATH BY DEMENTIA DIAGNOSIS AND RACE/ETHNICITY

**DOI:** 10.1093/geroni/igac059.1400

**Published:** 2022-12-20

**Authors:** Olga Jarrín, Haiqun Lin, Bei Wu, Paul Duberstein, Maria Lopez, Anum Zafar

**Affiliations:** Rutgers, The State University of New Jersey, New Brunswick, New Jersey, United States; Rutgers University, Newark, New Jersey, United States; New York University, New York, New York, United States; Rutgers School of Public Health, Piscataway, New Jersey, United States; Rutgers, The State University of New Jersey, New Brunswick, New Jersey, United States; Rutgers, The State University of New Jersey, New Brunswick, New Jersey, United States

## Abstract

Timing of hospice referral is critical to patient’s and family’s quality of life and satisfaction with hospice care. This study examined racial/ethnic variation in hospice use and factors associated with death at home among Medicare beneficiaries aged 50 years and older who died in 2018 (n=1,998,282). Hospice use was most frequent among non-Hispanic white beneficiaries (55.8%) followed by Hispanic (46.2%), Asian American (44.5%), Black (42.7%), and American Indian (42.1%) beneficiaries. Among decedents diagnosed with dementia, initiation of hospice prior to the last day of life was significantly associated with death at home among all racial/ethnic groups: non-Hispanic white (OR=2.62), Black (OR=2.04), Hispanic (OR=2.17), Asian American (OR=2.54), and American Indian (OR=2.76). Among decedents not diagnosed with dementia, initiation of hospice prior to the last day of life was less strongly associated with death at home: white (OR=1.33), Black (OR=0.83), Hispanic (OR=1.32), Asian American (OR=1.69), and American Indian (OR=1.56).

